# Sequencing saturation does not uniquely determine molecular recovery in UMI transcriptomics

**DOI:** 10.1093/bioinformatics/btag593

**Published:** 2026-08-08

**Authors:** Gavin W Wilson, Sangeetha N Kalimuthu, Jonathan C Yeung

**Affiliations:** Latner Thoracic Research Laboratories, University Health Network, Toronto, Ontario, Canada; Department of Surgery, University of Toronto, Toronto, Ontario, Canada; Department of Laboratory Medicine and Pathobiology, University of Toronto, Toronto, Ontario, Canada; Department of Laboratory Medicine and Pathobiology, University of Toronto, Toronto, Ontario, Canada; Laboratory Medicine Program, University Health Network, Toronto, Ontario, Canada; Latner Thoracic Research Laboratories, University Health Network, Toronto, Ontario, Canada; Department of Surgery, University of Toronto, Toronto, Ontario, Canada; Department of Medical Biophysics, University of Toronto, Toronto, Ontario, Canada

## Abstract

**Motivation:**

Accurate sequencing depth planning for UMI-based transcriptomic experiments currently relies on heuristic metrics such as reads per cell and sequencing saturation. However, sequencing saturation cannot uniquely determine molecular recovery because the relationship between these quantities depends on amplification heterogeneity.

**Results:**

Here we present NB-Lib, a modeling framework based on a zero-truncated negative binomial representation of reads per molecule that jointly estimates amplification heterogeneity and library complexity from transcriptomic libraries. Across 150 single-cell and spatial transcriptomic datasets, NB-Lib accurately reconstructs sequencing saturation curves and predicts sequencing depth requirements from shallow pilot sequencing experiments. We show that amplification heterogeneity and library complexity define a compact parameterization linking sequencing depth, saturation, and molecular recovery across transcriptomic platforms and explain substantial variation in sequencing cost and recovery between samples and technologies. Finally, we demonstrate that molecular recovery directly determines the reproducibility of low-abundance gene detection. Together, these results establish a unified framework for interpreting sequencing saturation, molecular recovery, and sequencing efficiency across UMI-based transcriptomic technologies.

**Availability:**

The scdepth package is available at https://github.com/gwlab-ca/scdepth. Code needed to reproduce the analyses presented in this work is available at https://github.com/gwlab-ca/scdepth_manuscript. A selection of raw data and all the post-processed data is available at https://doi.org/10.5281/zenodo.15518941.

## 1 Introduction

Unique molecular identifier (UMI)-based single-cell and spatial transcriptomic technologies enable quantitative profiling of gene expression across thousands to millions of cells or spatial barcodes per experiment (Ståhl *et al.* 2016, Zheng *et al.* 2017, Rosenberg *et al.* 2018, Oliveira *et al.* 2025). These platforms support applications ranging from hypothesis-driven studies to large-scale reference and clinical atlases. As these technologies evolve and are applied to increasingly diverse sample types, preparation methods, and sequencing platforms, determining sequencing depth remains a practical challenge (Svensson *et al.* 2017).

Current sequencing guidelines rely on heuristic metrics such as reads per cell, spatial barcode depth, and sequencing saturation (a measure of read redundancy). However, these metrics do not directly reflect molecular recovery (hereafter referred to as recovery), defined as the fraction of detectable molecules in the library that have been sequenced at least once. Libraries with similar barcode depths can exhibit different saturation values due to differences in library complexity and different recovery levels due to differences in amplification heterogeneity. This complicates comparisons between samples and limits comparisons across methods and platforms. Importantly, sequencing depth and saturation are often used to guide sequencing decisions but do not directly reflect recovery.

The reads-per-molecule (RPM) distribution within a library reflects amplification heterogeneity. The mean of the RPM distribution determines the saturation, and the shape of this distribution reflects amplification heterogeneity. Methods such as preseq (Daley and Smith 2013) use the RPM distribution and an Empirical Bayes power-series estimator to extrapolate library complexity and estimate molecular counts at a given read depth. However, preseq does not provide explicit parameters describing amplification heterogeneity or the relationship between saturation and recovery.

The RPM histogram has been previously modeled using the zero-truncated negative binomial distribution (ZT-NB) by BUTTERFLY (Hilbe 2014, Gustafsson *et al.* 2021) to handle molecules with zero reads being unobserved. The ZT-NB fit is then used to estimate the fraction of missing molecules for individual genes to better understand the “pooled amplification paradox.” The negative binomial distribution has also been used to model UMI count data in scRNA-seq datasets and to identify highly variable genes (Andrews and Hemberg 2019, Hafemeister and Satija 2019, Svensson 2020). However, the parameters of the ZT-NB fit, their association with amplification heterogeneity, their relationship to saturation, and their application to sequencing depth planning have not been systematically explored.

In this work, we show that the global reads-per-molecule (RPM) histogram of a UMI-based transcriptomic library can be modeled using a zero-truncated negative binomial distribution to estimate two interpretable parameters describing amplification heterogeneity and library complexity. We integrate these parameters into a library-level model, NB-Lib, that predicts the number of detected molecules, saturation, and recovery at a given sequencing depth, enabling accurate prediction of sequencing requirements from pilot sequencing experiments and providing a quantitative basis for sequencing depth planning. Using this framework, we demonstrate that recovery is not equivalent to saturation and that these quantities can require substantially different sequencing depths.

To evaluate this approach, we developed a deterministic nested downsampling framework and applied it to a cohort of 150 transcriptomic datasets spanning multiple scRNA-seq chemistries (Zheng *et al.* 2017), tissue types, and spatial transcriptomics platforms, including Visium (Ståhl *et al.* 2016) and Visium HD (Oliveira *et al.* 2025). The cohort also included a Parse Biosciences WT library, which was analyzed separately because of its distinct library preparation workflow (Supplemental Note 2, available as supplementary data at *Bioinformatics* online). Using this framework, we show that amplification heterogeneity and library complexity can be used to benchmark library preparation methods and sequencing chemistries. Finally, we demonstrate that recovery directly influences the detection of single-molecule genes, which strongly affects barcode stability across repeated downsampling experiments. Together, these results establish a unified framework for sequencing depth planning and for interpreting library complexity and amplification heterogeneity across diverse UMI-based transcriptomic technologies, allowing users to make data-driven decisions about when additional sequencing will yield meaningful biological signal versus redundant reads.

## 2 Methods

All analyses in this study were performed using the scdepth command-line package. The package provides commands for baseline ZT-NB fitting, limited saturation analyses, and downstream stability analyses. The outputs of these commands constitute the complete set of intermediate files used to generate the manuscript figures. Jupyter notebooks are provided that reproduce all figures directly from these outputs. A Snakemake (Mölder *et al.* 2021) workflow is also provided that runs all of the analyses necessary for the manuscript.

### 2.1 NB-Lib framework

**Figure 1:**
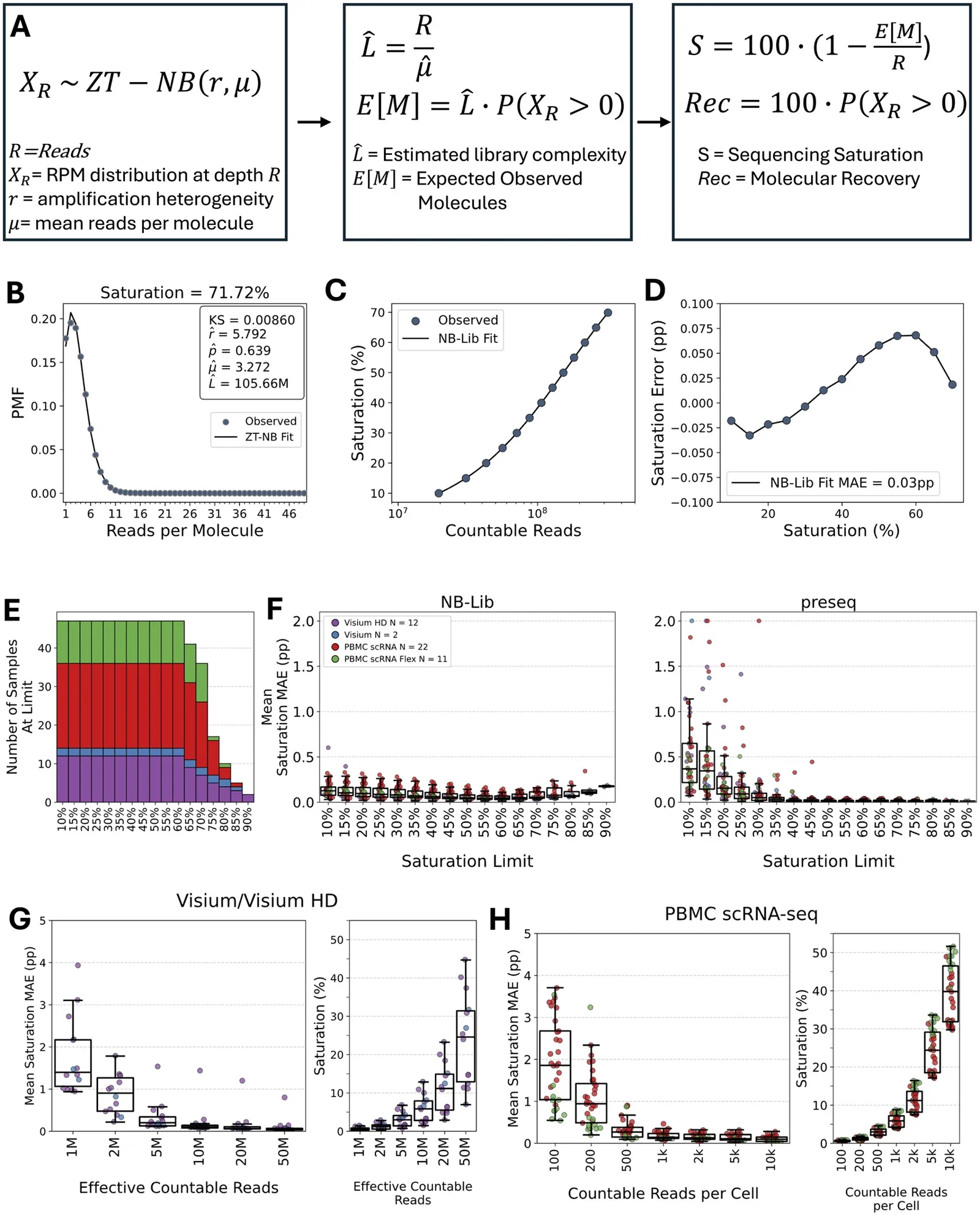
NB-Lib accurately reconstructs the saturation curve across sequencing depths. (A) NB-Lib models RPM distributions using a zero-truncated negative binomial framework to estimate library complexity, molecular recovery, and sequencing saturation. For detailed derivations see Supplementary Note 1, available as supplementary data at *Bioinformatics* online. (B–D) Example NB-Lib fit on a representative Visium HD sample. (B) A zero-truncated negative binomial distribution was fit to a representative Visium HD sample (Visium_HD_Human_Lung_Cancer_HD_Only_Experiment1) with 71.72% saturation. Estimated parameters are indicated in the box. Observed values are shown as dots and the fitted distribution as a line. (C, D) NB-Lib was used to predict the countable reads required to reach saturation values ranging from 10% to 70% in steps of 5%. scdepth was then used to generate nested downsampling steps at the predicted depths to determine the observed saturation values. Observed values are shown as dots and the model fit as a solid line, as indicated in the legend. Saturation versus countable reads is shown in **(C)**, and the prediction error (percentage points) plotted versus saturation in (D). (E, F) Downsampling validation across libraries. Exemplar libraries with at least 60% saturation were downsampled to a grid of saturation points starting at 10% and ending before the full-depth saturation. Ten independent nested downsampling seeds were processed per sample such that lower saturation points are subsets of higher saturation points. The number of samples at each downsampling step is shown in (E) and colored as indicated by the legend in (F). At each saturation limit, the RPM histogram was calculated and used to fit NB-Lib (left) and preseq (right). (F) The mean saturation mean absolute error (MAE) across seeds in percentage points (pp) is shown as one dot per sample and colored as indicated by the legend. Samples are summarized as boxplots and the MAE was capped at 2 pp. **(**G, H) Pilot depth extrapolation. The samples were further downsampled to pilot depth levels using the same nested downsampling scheme with ten independent seeds, and the full limited saturation curves used in (F) were extrapolated from these pilot points. (G) For Visium and Visium HD datasets, effective countable reads were calculated as the indicated countable reads multiplied by the fraction of the slide covered with tissue as estimated by a pathologist. Left, the mean saturation MAE across ten downsampling seeds and right, the observed saturation values. Each dot represents a sample and samples are summarized as boxplots **(**H) For the PBMC scRNA-seq datasets, countable reads per cell were calculated using the number of cells estimated by Drop-seq and basic filtering (see Methods) from the full depth dataset, multiplied by the indicated pilot depth. Left, the mean saturation MAE across ten downsampling seeds and right, the observed saturation values. Each dot represents a sample and samples are summarized as boxplots.

An overview of the NB-Lib framework is provided in Fig. 1A. Detailed derivations of the ZT-NB model and parameter estimation procedures are provided in Supplementary Note 1, available as supplementary data at *Bioinformatics* online.

### 2.2 Sample processing and cohorts

Detailed sample processing procedures for the single-cell RNA-seq, Visium, and Visium HD cohorts are provided in Supplemental Note 1, available as supplementary data at *Bioinformatics* online, and included samples are summarized in Table S1, available as supplementary data at *Bioinformatics* online. Processing and analysis of the Parse Biosciences WT library are described in Supplemental Note 2, available as supplementary data at *Bioinformatics* online.

### 2.3 scdepth framework

The scdepth framework was primarily developed in C++ and wrapped with Python. All analyses described below are implemented as command-line tools within scdepth. The scdepth framework is designed to enable efficient processing of large transcriptomic datasets and reproducible downsampling analyses.

To ensure consistent RPM histogram construction across datasets processed with different alignment tools, versions, and transcriptome references, reads were uniformly filtered to retain countable reads only, classified, and collapsed into barcode-gene-UMI tags using annotation aware methods (Supplemental Note 1, available as supplementary data at *Bioinformatics* online). Deterministic nested downsamping was implemented using barcode-specific random seeds and binomial sampling of the tags. This enabled reproducible reuse of read subsets across sequencing depths. Finally, directional UMI collapsing was used by default with alternative collapsing strategies implemented for comparison purposes (Supplemental Note 1, available as supplementary data at *Bioinformatics* online).

### 2.4 Dataset baseline saturation estimation

For samples with greater than 55% saturation, we used a two-step procedure to downsample to a 50% saturation baseline. First, we used the full-depth dataset and NB-Lib to estimate the number of countable reads required to achieve 50% saturation. The sample was then downsampled to this read depth, after which the countable reads required for 50% saturation were recalculated. Additional methods for sample processing are described in (Supplemental Note 1, available as supplementary data at *Bioinformatics* online).

## 3 Results

### 3.1 NB-Lib accurately models RPM distributions and sequencing saturation

Sequencing depth recommendations for UMI-based transcriptomic technologies remain largely heuristic because they depend on several experimental factors, including sample preparation quality, library chemistry, technology platform, and cell input for single-cell RNA-seq and tissue area for Visium and Visium HD. Suboptimal depth choices can lead to unnecessary sequencing costs for large-scale datasets or incomplete recovery in expensive assays. Moreover, sequencing depth and saturation are insufficient for cross-method comparisons because they do not uniquely determine molecular recovery.

To address these limitations, we modeled the reads-per-molecule (RPM) distribution using a zero-truncated negative binomial (ZT-NB) framework. This approach enables estimation of amplification heterogeneity and library complexity directly from RPM histograms without requiring sequencing-depth based curve fitting. The amplification heterogeneity ($\hat{r}$) and mean RPM ($\hat{µ}$) are derived from the ZT-NB fit. The total number of reads (R) equals the sum of reads across all molecules; therefore, the library complexity ($\hat{L}$) can be estimated as $\hat{L}=R/\hat{µ}$. With these parameters, the ZT-NB model provides a closed-form expression for the expected number of molecules observed at a given sequencing depth (Fig. 1A, see Supplemental Note 1, available as supplementary data at *Bioinformatics* online for derivation). The model also provides estimates of sequencing saturation defined as $100\cdot (1-\frac{E[M]}{R})$, where E[M] is the expected number of detected molecules and molecular recovery defined as $100\cdot P(X_{R}>0)$, the percentage of molecules expected to receive at least one read.

We developed NB-Lib to model the dependencies between sequencing depth, molecular recovery, and saturation, such that any one metric can be used to predict the other two. First, we demonstrate that our ZT-NB fits accurately model the RPM distribution at low to moderate saturation and then demonstrate that NB-Lib accurately predicts saturation curves from ultra-low sequencing depths.

To facilitate reproducible assessment of the NB-Lib model and comparisons with preseq, we developed scdepth, a deterministic and nested downsampling framework that enables systematic evaluation of sequencing depth by emitting detailed dataset- and barcode-level metrics, generating gene expression matrices, implementing the NB-Lib model, and enabling direct comparisons with preseq (Daley and Smith 2013) (see Methods). The NB-Lib model is independent of scdepth and can be implemented in any alignment or quantification workflow that produces RPM histograms. A key aspect of scdepth is that the framework operates directly on BAM files and is customizable for other UMI-based datasets. It also uses an indexed alignment cache for rapid downsampling analyses.

To evaluate ZT-NB and NB-Lib fits, we collected datasets with at least 60% saturation, comprising *N* = 33 peripheral blood mononuclear cell (PBMC) samples spanning diverse library chemistries and preparation methods, including the probe-based Flex assay. The PBMC cohort includes two biological replicates each with two technical replicates from Elz *et al.* (2026) for 10× Genomics v3.1, v4 and Flex chemistries (see Table S1, available as supplementary data at *Bioinformatics* online for individual 10× samples and figure usage). We further included 2 probe-based Visium FFPE samples and 12 probe-based Visium HD samples derived from FFPE, fixed frozen and fresh frozen tissues.

We first evaluated whether the ZT-NB model accurately captures RPM distributions and whether NB-Lib can predict saturation curves from limited sequencing depths. We selected a Visium HD lung sample with 71.7% saturation, for which the ZT-NB fit had a Kolmogorov–Smirnov (KS) statistic of 0.0086, indicating a close fit to the RPM distribution (Fig. 1B) (Wasserman 2010). Using NB-Lib, we estimated the number of countable reads required to achieve a range of saturation values and validated the predictions using scdepth. Across saturation values from 10% to 70%, the mean absolute error (MAE) was 0.03 percentage points (pp), indicating excellent agreement between predicted and observed values (Fig. 1C, D).

We next evaluated whether the ZT-NB parameters remain stable across sequencing depths. Using the cohort of samples with >60% saturation, we evaluated the robustness of the ZT-NB fit and NB-Lib fits to differing saturation. For each sample, we generated *N* = 10 independent nested downsampling seeds at saturation limits ranging from 10% to below the maximum saturation in steps of 5%. The number of samples at each limit is shown in Fig. 1E. At each limit, we fit the ZT-NB model to the RPM histogram and evaluated goodness-of-fit using the KS statistic. KS values remained less than < 0.01 across most values, indicating stable model fits, but increased gradually at extreme saturation values (Fig. S1A, available as supplementary data at *Bioinformatics* online). These increases in KS values were associated with the emergence of an excess population of low RPM molecules that became detectable at high saturation (Fig. S2, available as supplementary data at *Bioinformatics* online).

We then assessed whether these parameters enable accurate prediction of saturation curves. We benchmarked NB-Lib against preseq, a widely used method for sequencing depth estimation that also uses the RPM histogram for inference (Daley and Smith 2013). RPM histograms generated by scdepth were used as input for both NB-Lib and preseq to ensure that both methods were evaluated using identical data. Across the cohort, NB-Lib accurately reconstructed saturation curves, maintaining a mean absolute error (MAE) of <0.5 percentage points (pp) across the full range of evaluated saturation limits for nearly all samples (Fig. 1F; Fig. S2B, available as supplementary data at *Bioinformatics* online). preseq showed comparable prediction error at moderate sequencing depths but exhibited greater variability across datasets and sequencing depths and occasionally failed to generate predictions for a small number of seeds (Fig. 1F; Fig. S2C, D, available as supplementary data at *Bioinformatics* online).

The observed predictive stability of NB-Lib is consistent with the underlying parameter estimates. The amplification heterogeneity parameter ($\hat{r}$) and library complexity parameter ($\hat{L}$) were stable across low to moderate saturation values, with a mean percent error (MAE) of <10% relative to estimates at 50% saturation across the 10%–70% saturation range (Fig. S3A, B, available as supplementary data at *Bioinformatics* online). In contrast, complexity estimates derived using preseq (Fig. S3C, available as supplementary data at *Bioinformatics* online) exhibited a lower stability across sequencing depths. Although preseq produced similar complexity estimates for Visium and Visium HD datasets (mean percent differences <10%), its performance was markedly less consistent for PBMC scRNA-seq datasets, with larger discrepancies emerging at moderate to high saturation (Fig. S3D, available as supplementary data at *Bioinformatics* online). Finally, to assess whether prediction errors varied systematically across saturation values, we calculated the saturation prediction error at each saturation value for each limited-depth curve. NB-Lib predictions remained stable across saturation values, whereas preseq predictions derived from limited depths frequently exhibited elevated error at high saturation values (Fig. S4, available as supplementary data at *Bioinformatics* online).

Collectively, these results demonstrate that the ZT-NB model more robustly captures RPM distributions and yields stable parameter estimates across a broad range of sequencing depths.

To evaluate the feasibility of sequencing depth planning from pilot data, we next assessed whether NB-Lib can accurately predict saturation curves from ultra-low depths. For the Visium and Visium HD cohorts, we defined the effective countable reads as the target reads multiplied by the slide area covered by tissue, as assessed by an anatomical pathologist. We observed that the mean saturation MAE rapidly drops to a reliable level between 2M and 10M effective reads, which represents a saturation range between 0.3% and 13% (Fig. 1G). For the PBMC scRNA-seq cohort, reads were divided by the number of cells identified by emptyDrops (Lun *et al.* 2019) and basic filtering (See methods). We observed reliable between 500 and 1000 reads per cell which represents a saturation range between 2% and 16.5% (Fig 1H). One caveat of this analysis is that we used countable reads; however, these can be readily converted to sequencing reads by scaling with an estimated alignment rate. For scRNA-seq the alignment rate can vary substantially (∼40% to 80% across our full cohort of scRNA-seq samples, see Table S1, available as supplementary data at *Bioinformatics* online) while probe-based Visium, Visium HD, and flex assays tend to have high alignment rates (>90%).

We also analyzed a matched Parse Biosciences WT v3 PBMC library corresponding to four samples from the *Elz et al.* cohort (see Supplemental Note 2, available as supplementary data at *Bioinformatics* online for Parse WT library processing and analysis). Because the Parse WT v3 workflow uses combinatorial indexing, sample multiplexing, and dual priming with poly(A) and random-hexamer primers, it was analyzed separately using a dedicated preprocessing workflow. Nevertheless, the Parse WT v3 library exhibited similarly accurate ZT-NB fits and NB-Lib saturation predictions to the 10x Genomics libraries.

Finally, we examined the NB-Lib predicted saturation at manufacturer-recommended sequencing depths across an expanded cohort of 149 primary samples, including those analyzed above together with additional datasets from multiple cohorts (see Methods and Table S1, available as supplementary data at *Bioinformatics* online) (Elmentaite *et al.* 2021, Knight-Schrijver *et al.* 2022, Vázquez-García *et al.* 2022, Maitra *et al.* 2023, Elz *et al.* 2026). We observed substantial variability in the predicted saturation achieved at the recommended sequencing depths. Visium HD and Flex libraries ranged from approximately 10%–80% predicted saturation, whereas the scRNA-seq libraries ranged from approximately 20%–60% (Fig. S5, available as supplementary data at *Bioinformatics* online). These results demonstrate that fixed sequencing-depth recommendations can yield substantially different sequencing completeness across libraries, whereas NB-Lib provides library-specific sequencing depth estimates.

Taken together, these results demonstrate that NB-Lib enables accurate prediction of saturation curves from pilot sequencing experiments across both spatial transcriptomics and single-cell RNA-seq datasets.

### 3.2 Amplification heterogeneity and library complexity determine sequencing depth, recovery and saturation

Sequencing saturation alone does not uniquely determine molecular recovery because the relationship depends on amplification heterogeneity.

**Figure 2:**
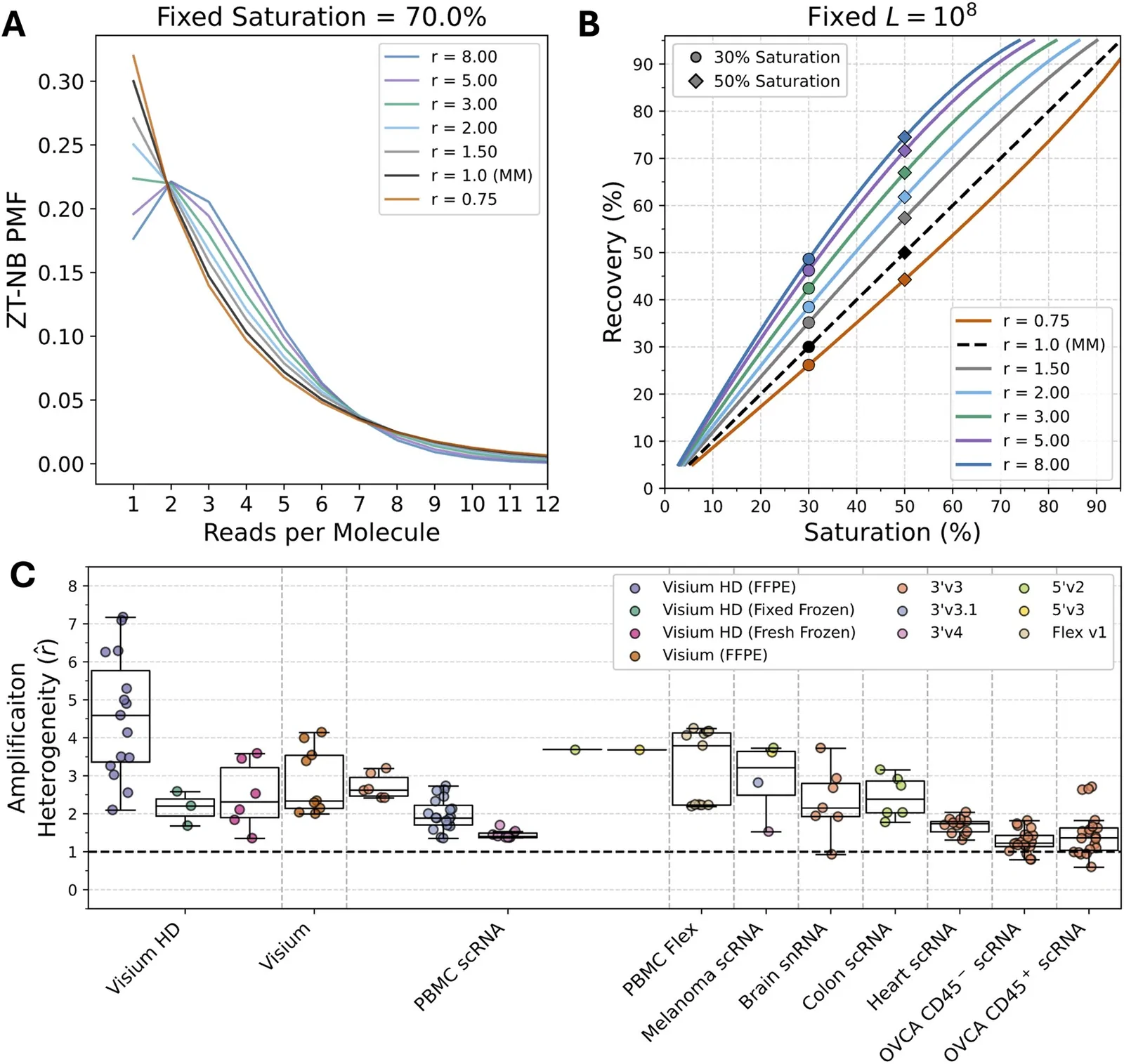
Amplification heterogeneity controls the relationship between saturation and recovery. (A) ZT-NB probability mass functions of reads per molecule for a fixed saturation (70%). Curves correspond to different values of the dispersion/amplification heterogeneity parameter r as indicated by the legend. (B) Recovery versus saturation expressed in percentages for a fixed library size $L=10^{8}$. Each curve corresponds to a different amplification heterogeneity value (r) as indicated by the legend. Points indicate 30% and 50% saturation values. The special case where *r* = 1 is indicated with a dashed line, where the NB-Lib function reduces to a Michaelis-Menten form and recovery is equal to saturation. (C) Predicted amplification heterogeneity ($\hat{r}$) from the ZT-NB model across the full cohort of samples. Samples are grouped by dataset and colored by library type as indicated in the legend. Vertical lines denote dataset boundaries and the horizontal lines indicate $\hat{r}=1$.

To illustrate this, we first examined how the amplification heterogeneity parameter (r) alters the shape of the ZT-NB probability mass function at fixed saturation (Fig. 2A). Lower values of r produced a heavier tail in the RPM distribution, which indicates greater amplification heterogeneity, whereas higher r values produced narrower distributions. Next, we examined how r affects the relationship between recovery and saturation using a fixed library size. When r = 1 the NB-Lib model reduces to a Michaelis-Menten form where saturation and recovery are equal. Increasing values of r > 1 led to higher recovery at a given saturation. For example, at 50% saturation r = 5 had ∼71.6% recovery (Fig. 2B). Values of r < 1 correspond to extreme amplification heterogeneity where recovery is lower than saturation; for example, at 50% saturation with r = 0.75, the recovery is 44.3%.

Finally, we examined the distribution of estimated amplification heterogeneity ($\hat{r}$) across our expanded cohort of 149 primary samples. We also included the matched Parse Biosciences WT v3 PBMC library from the *Elz et al.* cohort (see Supplementary Note 2, available as supplementary data at *Bioinformatics* online for Parse WT library processing and analysis). To ensure consistent parameter estimation across datasets, samples with saturation greater than 55% were downsampled to a 50% saturation baseline for subsequent analyses unless otherwise noted (see Methods). We found that $\hat{r}$ varied across platforms and within cohorts. For example, Visium HD FFPE samples exhibited the largest range of $\hat{r}$ values ranging from 2.1 to 7.2, while the remaining cohorts had $\hat{r}$ values between 0.78 and 4.2 (Fig. 2C).

These results demonstrate that amplification heterogeneity varies substantially across UMI-based transcriptomic technologies and directly influences the relationship between recovery and saturation.

### 3.3 Amplification heterogeneity and library complexity define a parameter space linking sequencing depth, recovery, and saturation across UMI-based transcriptomic technologies

Sequencing saturation alone does not uniquely determine molecular recovery because the relationship depends on amplification heterogeneity. To illustrate this, we first examined how the amplification heterogeneity parameter (r) alters the shape of the ZT-NB probability mass function at fixed saturation (Fig. 2A). Lower values of r produced a heavier tail in the RPM distribution, which indicates greater amplification heterogeneity, whereas higher r values produced narrower distributions. Next, we examined how r affects the relationship between recovery and saturation using a fixed library size. When r = 1 the NB-Lib model reduces to a Michaelis-Menten form where saturation and recovery are equal. Increasing values of r > 1 led to higher recovery at a given saturation. For example, at 50% saturation r = 5 had ∼71.6% recovery (Fig. 2B). Values of r < 1 correspond to extreme amplification heterogeneity where recovery is lower than saturation; for example, at 50% saturation with r = 0.75, the recovery is 44.3%.

Finally, we examined the distribution of estimated amplification heterogeneity ($\hat{r}$) across our expanded cohort of 149 primary samples. We also included the matched Parse Biosciences WT v3 PBMC library from the *Elz et al.* cohort (see Supplementary Note 2, available as supplementary data at *Bioinformatics* online for Parse WT library processing and analysis). To ensure consistent parameter estimation across datasets, samples with saturation greater than 55% were downsampled to a 50% saturation baseline for subsequent analyses unless otherwise noted (see Methods). We found that $\hat{r}$ varied across platforms and within cohorts. For example, Visium HD FFPE samples exhibited the largest range of $\hat{r}$ values ranging from 2.1 to 7.2, while the remaining cohorts had $\hat{r}$ values between 0.78 and 4.2 (Fig. 2C).

These results demonstrate that amplification heterogeneity varies substantially across UMI-based transcriptomic technologies and directly influences the relationship between recovery and saturation.

### 3.4 Amplification heterogeneity and library complexity define a parameter space linking sequencing depth, recovery, and saturation across UMI-based transcriptomic technologies

Amplification heterogeneity ($\hat{r}$) determines the relationship between recovery and saturation but does not fully determine the sequencing depth requirements, which also depend on the library complexity ($\hat{L}$). We therefore examined whether the combination of $\hat{r}$ and $\hat{L}$ defines a parameter space to compare samples across preparations and chemistries. To facilitate comparisons, we normalize $\hat{L}$ and sequencing depth (in countable reads) by the number of cells for scRNA-seq libraries or the tissue fraction for Visium and Visium HD libraries.

**Figure 3:**
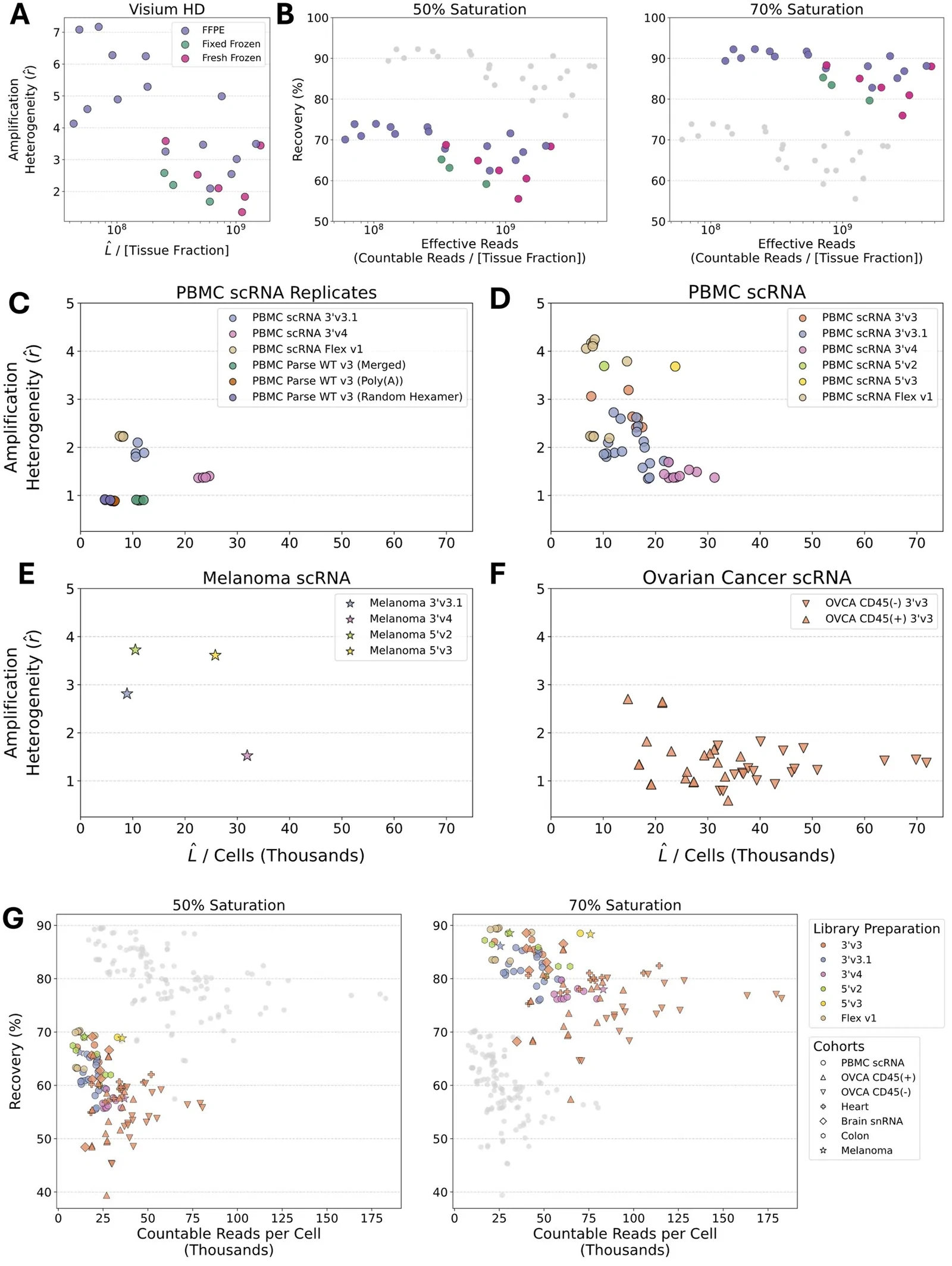
Amplification heterogeneity and library complexity determine the relationship between sequencing depth, saturation, and recovery. (A) The amplification heterogeneity parameter ($\hat{r}$) versus the library complexity normalized to pathologist determined tissue coverage fraction ($\hat{L}$) for Visium HD samples. Samples are colored by their tissue preparation method as indicated by the legend. (B) The recovery versus effective reads (Countable reads target multiplied by the fraction of tissue coverage) at 50% (left) and 70% (right) saturation for Visium HD samples. Samples are colored by their tissue preparation method as indicated by the legend. (C–F) The amplification heterogeneity parameter ($\hat{r}$) versus the library complexity normalized to the number of cells as determined by emptydrops and basic filtering (See Methods) for the indicated scRNA-seq cohorts. Samples are colored as per the legend in G. (G) The recovery versus effective reads (Countable reads per cell) at 50% (left) and 70% (right) saturation colored by the scRNA-seq cohort as indicated by the legend. Gray points show the corresponding values for the same samples at the other saturation target.

Across the Visium HD cohort, samples occupied a broad range of $\hat{r}$ and $\hat{L}$ values, indicating substantial variation in both amplification heterogeneity and library complexity (Fig. 3A). We next examined how these differences translated into sequencing depth requirements. For a fixed saturation target, samples occupying different regions of the parameter space exhibited large differences in recovery and depth requirements (Fig. 3B). For example, at 50% saturation, recovery varied between 55% and 74%, while the effective countable reads ranged from 60M and 2.2B, corresponded to 36M and 1.54B countable reads. We observed a similar trend for Visium FFPE samples where recovery varied between 62% and 70% and effective read cost varied between 200M and 2.7B, corresponding to 116M and 1.01B countable reads (Fig. S6A, B, available as supplementary data at *Bioinformatics* online). Taken together, these results demonstrate that sequencing depth requirements can vary by more than an order of magnitude across Visium HD and Visium samples, highlighting the limitations of heuristic depth recommendations.

We next examined whether amplification heterogeneity and library complexity distinguish scRNA-seq chemistries and biological samples. We applied the same analysis to four different scRNA-seq cohorts spanning multiple chemistries. We first examined the PBMC dataset from Elz *et al.* (2026) containing two biological replicates, each sequenced with two technical replicates across three chemistries (3′v3.1, 3′v4, Flex, and Parse WT v3). Within each chemistry $\hat{r}$ was highly consistent with a mean of 1.92 +/− 0.13 for v3-3.1, 1.36 +/− 0.02 for 3’v4 and 2.23 +/− 0.01 for the Flex, and 0.90 +/− 0.01 for the Parse WT v3 libraries (Fig. 3C). Likewise, the mean normalized $\hat{L}$ differed substantially between chemistries. The mean normalized $\hat{L}$ for 3’v4 (23 624 +/− 870) was approximately twice that of the 3’v3.1 (11 062 +/− 870), Flex (7911 +/− 302) and the Parse WT v3 (11 301 +/− 590) libraries. These results are consistent with the probe-based design of Flex libraries, which limits complexity, and confirm the substantially higher complexity of 3’v4 chemistry. However, this increased complexity was accompanied by greater amplification heterogeneity, as indicated by lower $\hat{r}$ values. The Parse WT v3 library exhibited the greatest amplification heterogeneity among the evaluated PBMC chemistries while maintaining accurate ZT-NB and NB-Lib fits (Supplemental Note 2, available as supplementary data at *Bioinformatics* online).

Similar trends were observed across the full PBMC cohort and a matched melanoma dataset, where the newer 3′-v4 and 5′-v3 chemistries exhibited higher normalized $\hat{L}$ values than earlier versions (Fig. 3D, E). Finally, we examined whether normalized $\hat{L}$ captures biological differences between cell types using dissociated ovarian cancer samples from Vázquez-García *et al.* (2022). In the ovarian cancer datasets, most CD45⁻ (predominantly tumour and fibroblasts) samples exhibited higher normalized $\hat{L}$ than the CD45^+^ (immune cells) samples.

The heart scRNA-seq cohort also exhibited higher normalized $\hat{L}$ than the colon scRNA-seq and brain snRNA-seq cohorts (Fig. S6C, available as supplementary data at *Bioinformatics* online). Collectively, these observations suggest that amplification heterogeneity and library complexity capture both chemistry-specific and biological differences across scRNA-seq datasets.

Finally, across all scRNA-seq cohorts, we compared the recovery and sequencing depth required to reach 50% and 70% saturation (Fig. 3G). At the same saturation level, recovery varied dramatically, ranging from 39% to 70% at 50% saturation and 57%–90% at 70% saturation. The sequencing depth required to achieve these saturation targets also varied widely, ranging from 8000–80 000 reads per cell at 50% saturation and 17 000–182 000 reads per cell at 70% saturation. Thus, at the same saturation target, sequencing depth requirements varied by approximately an order of magnitude, while recovery varied by more than 30 percentage points. These observations are consistent with our Visium and Visium HD analyses, demonstrating that heuristic sequencing depth recommendations can lead to substantially different recovery and saturation outcomes.

Together, these results show that amplification heterogeneity and library complexity define a parameter space that explains differences in sequencing depth and recovery across UMI-based transcriptomic technologies.

#### 3.4.1 Spliced molecules have reduced amplification heterogeneity and higher recovery than ambiguous and unspliced classes

Our downsampling framework also quantifies spliced, unspliced, and ambiguous molecules and generates RPM histograms for each class (La Manno *et al.* 2018, He *et al.* 2023, Hjörleifsson *et al.* 2024). We therefore tested whether amplification heterogeneity differs across these read classes in non-probe-based scRNA-seq datasets where spliced information is retained. A ZT-NB distribution was fit to the RPM histogram for each read class. Across nearly all datasets, spliced molecules exhibited lower amplification heterogeneity, reflected by higher $\hat{r}$ values, compared with ambiguous and unspliced molecules (Fig S7A, B, available as supplementary data at *Bioinformatics* online).

When aggregated across samples, these differences in amplification heterogeneity translated into significantly higher recovery for spliced molecules relative the global recovery (two-tailed Wilcoxon signed-rank test, *P* = 6.36 × 10^−19^, median difference = +14.28 pp, Fig. S7C, available as supplementary data at *Bioinformatics* online). In contrast, unspliced molecules exhibited slightly lower recovery than the global estimate (*P* = 1.99 × 10^−5^ and a median difference = −0.770 pp), while ambiguous molecules showed a larger reduction in recovery (*P* = 5.83 × 10^−19^ and a median difference of −0.575 pp).

Together, these results indicate that amplification heterogeneity varies systematically across molecule classes, suggesting that molecules within a library are not exchangeable with respect to amplification efficiency.

### 3.5 Recovery affects gene detection stability across downsampling seeds

The reproducibility of gene detection across repeated sequencing experiments has not been systematically evaluated. To address this, we quantified gene detection stability by performing repeated independent downsampling experiments at fixed sequencing depths. To assess this, we selected Visium HD and PBMC scRNA-seq samples with sufficient sequencing depth, defined as having at least 1.5× the countable reads required to reach 70% recovery (corresponding to at most ∼64% of the available countable reads) ensuring that downsampling captured meaningful variation in gene detection stability across recovery levels. This retained *N* = 10 Visium HD samples from FFPE and fresh frozen tissues and *N* = 28 PBMC samples generated using 3′v3, 3′v3.1, and Flex v1 chemistries.

**Figure 4:**
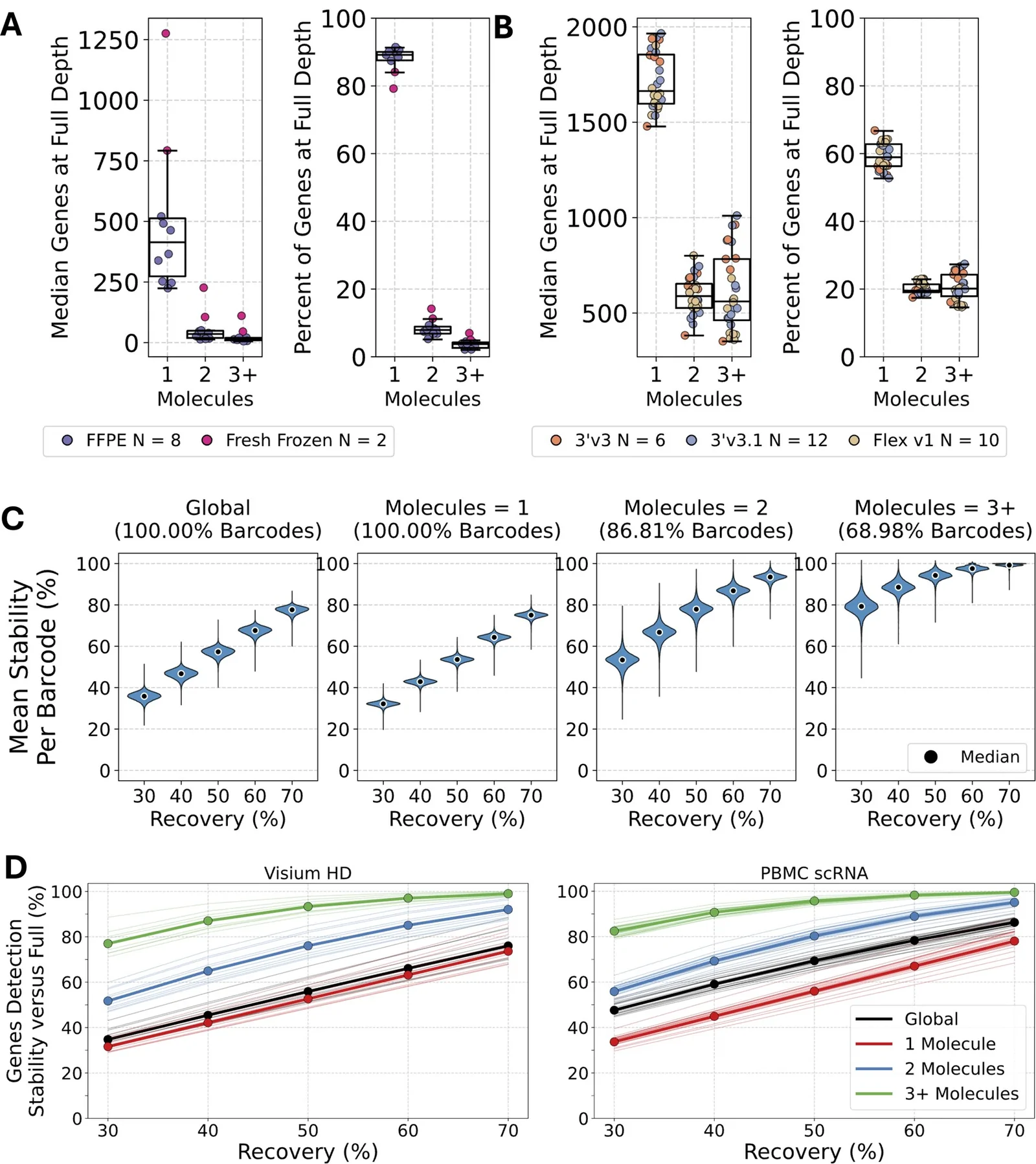
Gene detection stability is driven by low-abundance (single-molecule) genes. Samples with at least 1.5× more countable reads than required to achieve 70% molecular recovery (as estimated by NB-Lib) were selected. For Visium HD, 16 × 16 μm bins that passed filtering were used; for PBMC scRNA-seq, only filtered cells were included (see Methods). (A, B) Median number of genes (left) and percent of total genes (right) detected at full depth, grouped by molecule count class, for Visium HD (A) and PBMC scRNA-seq (B). Each point represents a sample, and distributions are summarized with boxplots. (C) Example Visium HD sample showing gene detection stability across barcodes (violin plots). (D) Gene detection stability as a function of recovery. For each barcode, stability was defined as the fraction of genes detected at full depth that were also detected after downsampling, averaged across 10 seeds. For each sample, stability was summarized as the median across all barcodes. Genes were stratified by molecule count at full depth (1 molecule, 2 molecules, and 3+ molecules). Barcodes were only retained if they had at least 10 unique gene counts for a given class. Thin lines indicate individual samples, while thick lines and points indicate the median across samples.

For Visium HD samples, barcodes were aggregated into 16 × 16 µm bins (8 × 8) and those passing filtering were retained (see Methods). For PBMC scRNA-seq samples, barcodes passing cell filtering were retained (see Methods). Low-abundance genes are expected to exhibit the greatest stochastic variability during downsampling. Accordingly, genes were stratified by their molecule count at full sequencing depth into four classes: genes supported by 1 molecule, 2 molecules, 3 or more molecules, and a global profile. Across both datasets, genes supported by a single molecule represented the largest fraction of detected genes in Visium HD and were strongly enriched in PBMC scRNA-seq datasets (Fig. 4A, B).

Each sample was then downsampled to recovery levels of 30, 40, 50, 60, and 70% using ten independent downsampling seeds. Gene detection stability was defined for each barcode as the fraction of genes detected at full depth that were also detected after downsampling, averaged across seeds and summarized as the median across barcodes (Fig 4C). Single-molecule genes exhibited the lowest stability, with detection closely tracking the downsampled recovery level in both Visium HD and PBMC scRNA-seq datasets (Fig. 4D). In contrast, genes supported by two or more molecules were substantially more stable across recovery levels. As single-molecule genes represent the largest fraction of detected genes, global gene detection stability closely followed the single-molecule class in Visium HD. In PBMC scRNA-seq samples, global stability was approximately 10% higher, reflecting the larger fraction of genes supported by two or more molecules.

We assessed how recovery influences the stability of common downstream analysis steps used in scRNA-seq and Visium HD workflows. Briefly, we used Scanpy to identify highly variable genes (HVGs), compute principal components, construct a K-nearest neighbor (KNN) graph, and perform Leiden clustering at three resolution parameters (see Methods). First, we evaluated the reproducibility of HVG selection across recovery levels by calculating the median pairwise Jaccard similarity between HVG sets obtained from independent downsampling seeds. In Visium HD samples, HVG similarity increased with recovery but varied substantially at lower recovery levels, whereas PBMC samples showed relatively stable HVG sets across all recovery values (Fig. S8A, available as supplementary data at *Bioinformatics* online). Next, we assessed the stability of the KNN graph by calculating the median pairwise Jaccard similarity of nearest neighbors for the same barcode across downsampling seeds, summarized as the median across barcodes. KNN similarity increased with recovery but remained lower than HVG similarity, particularly for Visium HD samples, reaching approximately 0.2, whereas PBMC samples showed higher stability, reaching approximately 0.4 (Fig. S8B, available as supplementary data at *Bioinformatics* online). Finally, we evaluated clustering stability by calculating the adjusted Rand index (ARI) between cluster assignments from different downsampling seeds. Leiden clustering was relatively stable across recovery levels, with ARI values of ∼0.5 for Visium HD samples and ∼0.75 for PBMC scRNA-seq samples (Fig. S8C, D, available as supplementary data at *Bioinformatics* online). Together, these results indicate that downstream clustering results remain comparatively stable across recovery levels while HVGS and KNN stability is more affected by recovery.

Collectively, these results demonstrate that gene detection reproducibility is largely driven by low-abundance genes supported by a single molecule, whose detection depends strongly on sequencing recovery.

## 4 Discussion

Accurate sequencing depth planning is critical for the design of UMI-based transcriptomic experiments, and dataset-level metrics are essential for comparing samples across preparation methods, platforms and chemistries. Current sequencing guidelines primarily rely on heuristic metrics such as reads per cell or spatial barcode and use sequencing saturation as a completeness metric. However, these guidelines can lead to libraries being under-sequenced, leaving recoverable molecules undetected, or over-sequenced, increasing costs with a marginal increase in recovery. Importantly, these metrics do not directly reflect the fraction of molecules that have been observed at least once. In this work we show that sequencing saturation alone does not uniquely determine recovery. Instead, amplification heterogeneity and library complexity jointly determine how sequencing depth translates into recovered molecules. By modeling the global reads-per-molecule (RPM) histogram using a zero-truncated negative binomial (ZT-NB) distribution, we derive two interpretable parameters that capture these effects and integrate them into a library-level model (NB-Lib) that predicts sequencing saturation and recovery across depths.

Across a diverse cohort of spatial and single-cell RNA-seq datasets, we show that the ZT-NB model provides a stable and accurate description of RPM distributions and that NB-Lib can accurately reconstruct sequencing saturation curves from limited sequencing depths. These analyses were enabled by the scdepth framework, which performs reproducible nested downsampling to any read fraction. Using this framework, we show that pilot sequencing experiments can reliably estimate sequencing depth requirements using only a small fraction of the reads typically recommended for these assays. For Visium and Visium HD datasets, pilot experiments were feasible with approximately 5–10 million countable reads per slide, scaling with tissue coverage. For scRNA-seq datasets, pilot experiments were feasible at approximately 500–1000 countable reads per cell, although the required number of sequenced reads depended on alignment rates. Together, these results demonstrate that pilot sequencing experiments can provide a practical strategy for calibrating sequencing depth requirements across diverse transcriptomic platforms.

The amplification heterogeneity ($\hat{r}$) and library complexity ($\hat{L}$) parameters inferred from the ZT-NB fits also provide a framework for comparing UMI-based transcriptomic libraries across preparation methods, chemistries and platforms. For example, we show that 3′ v4 libraries exhibit nearly twice the library complexity of 3′ v3.1 and Flex libraries. More broadly, these parameters define the relationship between recovery, saturation and sequencing depth, such that optimizing one metric inevitably affects the others. Selecting an appropriate optimization metric is therefore not straightforward. In particular, depth-based metrics such as reads per cell are limited because they do not capture differences in library complexity, and in our analyses these metrics varied by more than an order of magnitude across samples. Together, amplification heterogeneity and library complexity define a compact parameterization of sequencing efficiency that links sequencing depth, saturation and recovery across UMI-based transcriptomic libraries.

One key observation is that reduced amplification heterogeneity (higher $\hat{r}$ values) leads to increased recovery relative to saturation. Although the difference between saturation and recovery in real datasets is typically modest, it can reach approximately 20% in some cases. As a result, optimizing sequencing depth using saturation alone can lead to substantial over-sequencing for libraries with relatively low amplification heterogeneity. These observations highlight the limitations of depth-based sequencing recommendations and demonstrate the importance of jointly considering recovery and saturation when planning sequencing depth.

Several limitations of the current framework should be noted. The ZT-NB model assumes that amplification heterogeneity can be approximated by a negative binomial process. In our analyses, we observed that at very high saturation (>80%) the ZT-NB fit begins to degrade due to the emergence of low-RPM molecules, which may represent a class of molecules that are difficult to sequence. However, within practical saturation ranges the ZT-NB fits remained stable and the NB-Lib framework produced accurate predictions. To further mitigate potential biases at very high saturation, we downsampled samples with >55% saturation to a baseline value of 50% for downstream analyses. In addition, the quality of the ZT-NB fits depends on factors such as sample quality, UMI collapsing accuracy and gene assignment procedures. Alternative processing pipelines or lower-quality datasets may therefore yield reduced fit quality. We further demonstrated that NB-Lib accurately models a matched Parse Biosciences WT v3 library, indicating that the framework extends beyond the 10x Genomics library preparations examined in the primary analyses. Within this high-quality PBMC dataset, parameter estimates and prediction accuracy were highly consistent across multiplexed samples and primer types. However, additional studies using more diverse datasets and library preparation workflows will be required to establish the generality of these observations. Future work may explore extensions of the model to incorporate additional sources of variability and evaluate its applicability to additional UMI-based assays.

Despite these limitations, the NB-Lib framework provides a unified approach for interpreting sequencing efficiency across UMI-based transcriptomic technologies. By directly linking sequencing depth, recovery and saturation, NB-Lib enables more principled sequencing depth planning than heuristic metrics alone. More broadly, this framework provides a foundation for evaluating sequencing efficiency across emerging transcriptomic technologies and for benchmarking new library preparation strategies.

## Supplementary Material

btag593_Supplementary_Data
