# Supplementary material for: Sequencing saturation does not uniquely determine molecular recovery in UMI transcriptomics
Source: Bioinformatics. 2026 Aug 8;42(8):btag593. doi: 10.1093/bioinformatics/btag593 (PMC13476072; doi:10.1093/bioinformatics/btag593)
Supplement: Supplementary file 1 [file btag593_supplementary_data.zip › Supplemental/Supplemental Figures.docx]

**Extended Data:**

**Table S1. Summary of datasets used in this study.**

Comprehensive list of all samples included in this study, including dataset source, assay type, and sample metadata. For each sample, the figures in which it was used are indicated. Quality control metrics include total reads, countable reads, full-depth saturation, ZT-NB and NB-Lib fit parameters, and the number of cells or tissue coverage (see Methods for metric definitions).

**Table S2. Summary of the PBMC Parse WT v3 dataset.**

The table summarizes the combined library, individual samples, and primer-specific sub-libraries, including the total reads, countable reads, sequencing saturation, estimated ZT-NB fit parameters, the estimated library complexity, and the NB-Lib saturation prediction error (MAE). Refer to **Supplemental Note 2** for details of the analysis.

**Figure S1**

**Figure S1**

**A**

**B**

**C**

**D**

**Seed level error reproducibility.**
The downsampling limits and seeds in **Figure 1D, E** were used. The left column shows Visium and Visium HD datasets and the right column shows the PBMC datasets.

**A.** The Kolmogorov-Smirnov statistic for the ZT-NB fit at the indicated saturation limits for the Visium/Visium HD datasets (left) and PBMC datasets (right). Each dot indicates a single downsampling seed with N = 10 seeds per sample and is overlaid on boxplots summarizing the distribution at each saturation limit.

**B.** The NB-Lib MAE in percentage points.

**C.** The preseq MAE in percentage points. The MAE was capped at 10 percentage points.

**D.** The percent of preseq seeds that failed to produce output.

**Figure S2**

**Figure S2. Elevated low reads-per-molecule species reduce ZT-NB fit quality at ultra-high depth.**

The ZT-NB PMF error versus reads per molecule at 50% saturation (top) and full depth (bottom) for Visium and Visium HD datasets (left) and scRNA-seq datasets (right). Points are colored as indicated in the legend. At 50% saturation, only a single downsampled seed is shown per dataset.

**Figure S3**

**Parameter drift relative to 50% saturation.**

The downsampling limits and seeds in Figure 1D,E were used. Parameter drift relative to 50% saturation for NB-Lib and preseq.

The mean percent difference across ten seeds between the saturation limited calculate and the 50% baseline calculation. **A**. The NB-Lib ZT-NB fit $\hat{r}$ and **B.** The NB-Lib ZT-NB $\hat{L}$. **C**. The maximum library depth ( $\hat{L}$) as predicted by preseq **D**. The percent difference between the NB-Lib $\hat{L}$ and preseq $\hat{L}$.

**Figure S4**

**Figure S4**

**Absolute error across saturation values for NB-Lib and preseq**

The downsampling limits and seeds in **Figure 1D,E** were used with samples that have at least 80% saturation at full depth (**Table S1**). Full curves for each saturation limit are colored according to the colorbar. The mean saturation absolute error across ten seeds for each point on the curve is plotted on the y-axis versus the saturation point on the x-axis. NB-Lib fits (left) and preseq fits (right). Each row corresponds to a sample as indicated on the plot. The preseq MAE is capped at 2 pp.

**Figure S5**

**Figure S5. Model-predicted saturation at manufacturer-recommended sequencing depths.**

For Visium HD libraries, the recommended countable reads were calculated from the manufacturer recommendations of 275 million (FFPE), 500 million (Fixed Frozen), and 750 million (Fresh Frozen) reads, adjusted by the estimated alignment rate and the pathologist-quantified tissue area. For Flex v1 libraries, the recommended countable reads were calculated as 10,000 reads per cell multiplied by the estimated alignment rate. For the remaining scRNA-seq libraries, the recommended countable reads were calculated as 20,000 reads per cell multiplied by the estimated alignment rate. The boxplots show the NB-Lib predicted sequencing saturation at these manufacturer-recommended sequencing depths.

**Figure S6**

**A**

**C**

**B**

**Figure S6. Amplification heterogeneity and library complexity determine the relationship between sequencing depth, saturation, and recovery (additional samples)**

**A.** The amplification heterogeneity parameter ($\hat{r}$) versus the library complexity normalized to pathologist determined tissue coverage fraction ($\hat{L}$) for Visium samples. Samples are colored by their tissue preparation method as indicated by the legend.

**B.** The recovery versus effective reads (Countable reads target multiplied by the fraction of tissue coverage) at 50% (left) and 70% (right) saturation for Visium samples. Samples are colored by their tissue preparation method as indicated by the legend.

**C**. The amplification heterogeneity parameter ($\hat{r}$) versus the library complexity ($\hat{L}$) normalized to the number of cells as determined by emptydrops and basic filtering (See Methods). ScRNA-seq samples are colored by their cohort as indicated by the legend.

.

**Figure S7**

**A**

**B**

**C**

**Figure S7 Amplification heterogeneity across molecule classes.**

Molecules were assigned to one of three classes (spliced, unspliced, ambiguous) or the combined global class (total). Separate ZT-NB fits were performed for each class.

**A**. The distribution of $\hat{r}$ parameters for each scRNA-seq sample is shown as dots and summarized with boxplots.

**B**. The distribution of $\hat{r}$ parameters for each PBMC scRNA-seq library chemistry is shown as dots and summarized with boxplots.

**C**. The difference between the class recovery and the global recovery for spliced (first column), unspliced (second column) and ambiguous (third column) molecules. The median difference and P value calculated by a two-sided Wilcoxon signed-rank test are indicated as insets in each plot.

**Figure S8**

**A**

**B**

**C**

**D**

**Figure S8. Clustering is stable across molecular recoveries while highly variable genes and local neighbourhoods can vary.**

Barcodes for Visium HD 16 x 16 $\mu$m bins (8 x 8) that have passed filtering (see Methods) and PBMC barcodes that passed cell filtering (see Methods) were used for these analyses. The same samples were used in this analysis as **Fig. 4**.

**A**. The median pairwise Jaccard index between highly variable genes (HVG, N = 2,000) across ten downsampling seeds for Visium HD samples (left) and PBMC samples (right). Thick lines and dots indicate the median for each sample group as indicated in the legend and thin colored lines indicate individual samples.

**B**. The median pairwise Jaccard index between K-nearest neighbour graphs constructed using the HVGs, 30 principal components, and 30 nearest neighbours across ten downsampling seeds for Visium HD samples (left) and PBMC samples (right). Thick lines and dots indicate the median for each sample group as indicated in the legend and thin colored lines indicate individual samples.

**C, D**. The median pairwise adjusted Rand index across ten downsampling seeds for Leiden clustering with the indicated resolution parameter for **C**. Visium HD samples and **D**. PBMC samples. Thick lines and dots indicate the median for each sample group as indicated in the legend and thin colored lines indicate individual samples.

**Figure S9**

**A**

**B**

**C**

**D**

**E**

**F**

**G**

**Figure S9. Evaluation of NB-Lib on a Parse Biosciences WT v3 PBMC library.**

**A**. A zero-truncated negative binomial (ZT-NB) distribution was fit to the Parse WT v3 library (55.3% sequencing saturation). Estimated parameters are shown in the inset. Observed RPM frequencies are shown as dots and the fitted distribution as a solid line.

**B**. NB-Lib was used to predict the number of countable reads required to achieve saturation values from 10% to 55% (8 evenly spaced levels). scdepth was then used to generate nested downsampling fractions at the predicted read depths, and the observed saturation values were compared with the NB-Lib predictions. Observed values are shown as dots and the predicted saturation curve as a solid line.

**C-D**. The library was downsampled to saturation limits ranging from 10% to the full-depth saturation. Ten independent nested downsampling seeds were generated such that lower saturation fractions were subsets of higher saturation fractions. At each saturation limit, the RPM histogram was fit with the ZT-NB model. C. KS statistic of the ZT-NB fit. D. Saturation mean absolute error (MAE) in percentage points (pp). Lines indicate the median across seeds and dots indicate individual seeds.

**E-G**. The library was further downsampled to pilot sequencing depths using the same nested downsampling scheme with ten independent seeds. Limited-saturation curves were estimated from each pilot dataset and extrapolated to full depth. Countable reads per cell were calculated using the number of cells identified by EmptyDrops after quality-control filtering (see Methods). E. KS statistic of the ZT-NB fit. F. Saturation MAE (pp). G. Saturation as a function of countable reads per cell. Lines indicate the median across seeds and dots indicate individual seeds.

**Figure S10**

**A**

**B**

**C**

**D**

**E**

**F**

**Figure S10. Comparison of PBMC Parse Biosciences WT v3 sub-libraries.**

The full Parse WT v3 library was downsampled to sequencing saturations ranging from 10% to 55% in 5% increments. Reads were partitioned by sample and primer type (Merged, Poly(A), or Random Hexamer). Samples are colored according to the legend. The “All” sample includes the full multiplexed library.

**A.** Reads allocated to each sample within the indicated primer type (y-axis) versus the total countable reads for the full library (x-axis).

**B.** NB-Lib predicted saturation (solid line) compared with the empirically observed saturation (points) for each sample.

**C.** Kolmogorov-Smirnov (KS) statistic of the ZT-NB fit across the sub-libraries.

**D.** Estimated amplification heterogeneity ($\hat{r}$) across the sub-libraries.

**E.** the library complexity ($\hat{L}$) normalized to the number of cells as determined for each sample by EmptyDrops and basic filtering (See Methods). For the “All” sample the cell counts were summed across samples.

**F.** Saturation mean absolute error (MAE) in percentage points (pp) across the sub-libraries.
