# Supplementary material for: Sequencing saturation does not uniquely determine molecular recovery in UMI transcriptomics
Source: Bioinformatics. 2026 Aug 8;42(8):btag593. doi: 10.1093/bioinformatics/btag593 (PMC13476072; doi:10.1093/bioinformatics/btag593)
Supplement: Supplementary file 1 [file btag593_supplementary_data.zip › Supplemental/Supplemental Note 1.docx]

**Derivation of library complexity and molecular recovery from ZT-NB fits**

For all analyses in this manuscript, we use countable reads ($R$), which represent reads that can be assigned to an SAU class and used for UMI counting. The remaining reads are marked as uncountable ($B$), these include multi-mapped, antisense and unmapped reads. The mapping rate:

$$A=\frac{R}{R+B}$$

can be estimated at a given sequencing depth and used to estimate the sequenced reads $(T)$ from the number of countable reads ($R$)

T= $\frac{R}{A}$

We define sequencing saturation (referred to as saturation, $S$) as a function of countable reads $R$ and detected molecules $M$. This measure is directly related to the mean observed reads per molecules ($Mean RPM=R/M$).

$$S=100\left( 1-\frac{M}{R} \right)=100\left( 1-\frac{1}{Mean RPM} \right)$$

To model the relationship between sequencing depth and observed reads per molecule, we adopt a library-level framework based on the zero-truncated negative binomial (ZT-NB) distribution, referred to here as the NB-Lib model (Hilbe 2014). We selected the ZT-NB as an empirical model of the observed RPM distribution because it flexibly captures overdispersion while providing a useful analytical form for downstream derivations. We model the number of reads generated from each molecule using a negative binomial distribution with shape parameter $r$ and mean RPM $\mu$:

$X\sim NB(r,\mu)$.

From this, the probability that a molecule receives zero reads is:

$P\left( X=0 \right)={\binom{r}{r+\mu}}^{r}$(Equation 1)

A zero-truncated negative binomial (ZT-NB) fit to the observed reads-per-molecule histogram yields an estimated amplification heterogeneity (shape) parameter ($\hat{r}$) and mean ($\hat{\mu}$). If the library contains $L$ molecules, then the mean reads per molecule is

$\mu=\frac{R}{L}$ (Equation 2)

We estimate the library complexity as

$$\hat{L}=\frac{R}{\hat{\mu}}$$

Substituting Equation 2 into Equation 1 gives the expected fraction of molecules detected at countable read depth $R$

$$P\left( X>0 \right)=1-\left( \frac{r}{r+\frac{R}{L}} \right)^{r}$$

Multiplying by $L$ and substituting $r$ and $L$ with $\hat{r}$ and $\hat{L}$ gives the expected number of molecules

$$E\left[ M \right]=\hat{L}\left( 1-\left( \frac{\hat{r}}{\hat{r}+\frac{\hat{R}}{\hat{L}}} \right)^{\hat{r}} \right)$$

When implementing the ZT-NB fit, we use the equivalent SciPy negative binomial parameterization using $p=\frac{r}{r+\mu}$.

Finally, molecular recovery (denoted as $Rec$) at countable reads depth $R$ can be estimated as

$Rec=100\left( 1-\left( \frac{\hat{r}}{\hat{r}+\frac{R}{\hat{L}}} \right)^{\hat{r}} \right)$ (*Equation 3*)

Saturation can also be estimated as

$$S=100\left( 1-\frac{E[M]}{R} \right)=100\left( 1-\frac{1-\left( \frac{\hat{r}}{\hat{r}+\hat{u}} \right)^{\hat{r}}}{\hat{u}} \right)$$

The Michaelis–Menten form has previously been used to estimate gene and molecule abundances and identify highly variable genes from UMI-based transcriptomic datasets and arises here as a special case of the NB-Lib framework when $\hat{r}=1 ADDIN ZOTERO\_ITEM CSL\_CITATION \{"citationID":"UprNoTlQ","properties":\{"unsorted":false,"formattedCitation":"(Johnson and Goody 2011; Andrews and Hemberg 2019; Zhang et al. 2019; Simone et al. 2024)","plainCitation":"(Johnson and Goody 2011; Andrews and Hemberg 2019; Zhang et al. 2019; Simone et al. 2024)","noteIndex":0\},"citationItems":[\{"id":9165,"uris":["http://zotero.org/users/6668499/items/EWE37MXH"],"itemData":\{"id":9165,"type":"article-journal","abstract":"Nearly 100 years ago Michaelis and Menten published their now classic paper [Michaelis, L., and Menten, M. L. (1913) Die Kinetik der Invertinwirkung. Biochem. Z. 49, 333-369] in which they showed that the rate of an enzyme-catalyzed reaction is proportional to the concentration of the enzyme-substrate complex predicted by the Michaelis-Menten equation. Because the original text was written in German yet is often quoted by English-speaking authors, we undertook a complete translation of the 1913 publication, which we provide as Supporting Information. Here we introduce the translation, describe the historical context of the work, and show a new analysis of the original data. In doing so, we uncovered several surprises that reveal an interesting glimpse into the early history of enzymology. In particular, our reanalysis of Michaelis and Menten’s data using modern computational methods revealed an unanticipated rigor and precision in the original publication and uncovered a sophisticated, comprehensive analysis that has been overlooked in the century since their work was published. Michaelis and Menten not only analyzed initial velocity measurements but also fit their full time course data to the integrated form of the rate equations, including product inhibition, and derived a single global constant to represent all of their data. That constant was not the Michaelis constant, but rather Vmax/Km, the specificity constant times the enzyme concentration (kcat/Km \times E0).","citation-key":"johnsonOriginalMichaelisConstant2011","container-title":"Biochemistry","DOI":"10.1021/bi201284u","ISSN":"0006-2960","issue":"39","journalAbbreviation":"Biochemistry","page":"8264-8269","publisher":"American Chemical Society","source":"ACS Publications","title":"The Original Michaelis Constant: Translation of the 1913 Michaelis-Menten Paper","title-short":"The Original Michaelis Constant","volume":"50","author":[\{"family":"Johnson","given":"Kenneth A."\},\{"family":"Goody","given":"Roger S."\}],"issued":\{"date-parts":[["2011",10,4]]\}\}\},\{"id":9163,"uris":["http://zotero.org/users/6668499/items/T27ESPUJ"],"itemData":\{"id":9163,"type":"article-journal","abstract":"Most genomes contain thousands of genes, but for most functional responses, only a subset of those genes are relevant. To facilitate many single-cell RNASeq (scRNASeq) analyses the set of genes is often reduced through feature selection, i.e. by removing genes only subject to technical noise.We present M3Drop, an R package that implements popular existing feature selection methods and two novel methods which take advantage of the prevalence of zeros (dropouts) in scRNASeq data to identify features. We show these new methods outperform existing methods on simulated and real datasets.M3Drop is freely available on github as an R package and is compatible with other popular scRNASeq tools: https://github.com/tallulandrews/M3Drop.Supplementary data are available at Bioinformatics online.","citation-key":"andrewsM3DropDropoutbasedFeature2019","container-title":"Bioinformatics","DOI":"10.1093/bioinformatics/bty1044","ISSN":"1367-4803","issue":"16","journalAbbreviation":"Bioinformatics","page":"2865-2867","source":"Silverchair","title":"M3Drop: dropout-based feature selection for scRNASeq","title-short":"M3Drop","volume":"35","author":[\{"family":"Andrews","given":"Tallulah S"\},\{"family":"Hemberg","given":"Martin"\}],"issued":\{"date-parts":[["2019",8,15]]\}\}\},\{"id":9151,"uris":["http://zotero.org/users/6668499/items/GFJ3ZI2Z"],"itemData":\{"id":9151,"type":"article-journal","abstract":"Since its establishment in 2009, single-cell RNA sequencing (RNA-seq) has been a major driver behind progress in biomedical research. In developmental biology and stem cell studies, the ability to profile single cells confers particular benefits. Although most studies still focus on individual tissues or organs, the recent development of ultra-high-throughput single-cell RNA-seq has demonstrated potential power in characterizing more complex systems or even the entire body. However, although multiple ultra-high-throughput single-cell RNA-seq systems have attracted attention, no systematic comparison of these systems has been performed. Here, with the same cell line and bioinformatics pipeline, we developed directly comparable datasets for each of three widely used droplet-based ultra-high-throughput single-cell RNA-seq systems, inDrop, Drop-seq, and 10X Genomics Chromium. Although each system is capable of profiling single-cell transcriptomes, their detailed comparison revealed the distinguishing features and suitable applications for each system.","citation-key":"zhangComparativeAnalysisDropletBased2019","container-title":"Molecular Cell","DOI":"10.1016/j.molcel.2018.10.020","ISSN":"1097-2765","issue":"1","journalAbbreviation":"Molecular Cell","page":"130-142.e5","source":"ScienceDirect","title":"Comparative Analysis of Droplet-Based Ultra-High-Throughput Single-Cell RNA-Seq Systems","volume":"73","author":[\{"family":"Zhang","given":"Xiannian"\},\{"family":"Li","given":"Tianqi"\},\{"family":"Liu","given":"Feng"\},\{"family":"Chen","given":"Yaqi"\},\{"family":"Yao","given":"Jiacheng"\},\{"family":"Li","given":"Zeyao"\},\{"family":"Huang","given":"Yanyi"\},\{"family":"Wang","given":"Jianbin"\}],"issued":\{"date-parts":[["2019",1,3]]\}\}\},\{"id":9515,"uris":["http://zotero.org/users/6668499/items/ZRI8TDVF"],"itemData":\{"id":9515,"type":"article","abstract":"This study evaluates ten commercially available single-cell RNA sequencing (scRNA-seq) approaches across four technology groups: Emulsion-based kits from 10x Genomics and Fluent Biosciences; Microwell-based kits from Becton Dickinson, Honeycomb Technologies and Singerlon Technologies; Combinatorial-indexing kits from Parse Biosciences and Scale Biosciences; and a Matrigel-based kit from Scipio Biosciences. Peripheral blood mononuclear cells (PBMCs) from a single donor were used to assess analytical performance. Key features such as sample compatibility, cost, and experimental duration were also compared. Notably, superior analytical performance was demonstrated by the Chromium Fixed RNA Profiling kit from 10x Genomics, which uniquely features probe hybridization for transcript detection. Additionally, the Rhapsody WTA kit from Becton Dickinson provided a cost-effective balance of performance and expense per cell. With a rich dataset of 218,154 cells, this work provides a basis for differentiating commercial scRNA-seq technologies, which is intended to facilitate the effective application and further methodological development of single cell transcriptomics.","citation-key":"simoneComparativeAnalysisCommercial2024","DOI":"10.1101/2024.06.18.599579","language":"en","license":"© 2024, Posted by Cold Spring Harbor Laboratory. This pre-print is available under a Creative Commons License (Attribution-NonCommercial-NoDerivs 4.0 International), CC BY-NC-ND 4.0, as described at http://creativecommons.org/licenses/by-nc-nd/4.0/","note":"page: 2024.06.18.599579\backslash nsection: New Results","publisher":"bioRxiv","source":"bioRxiv","title":"Comparative Analysis of Commercial Single-Cell RNA Sequencing Technologies","URL":"https://www.biorxiv.org/content/10.1101/2024.06.18.599579v1","author":[\{"family":"Simone","given":"Marco De"\},\{"family":"Hoover","given":"Jonathan"\},\{"family":"Lau","given":"Julia"\},\{"family":"Bennet","given":"Hayley"\},\{"family":"Wu","given":"Bing"\},\{"family":"Chen","given":"Cynthia"\},\{"family":"Menon","given":"Hari"\},\{"family":"Au-Yeung","given":"Amelia"\},\{"family":"Lear","given":"Sean"\},\{"family":"Vaidya","given":"Samir"\},\{"family":"Shi","given":"Minyi"\},\{"family":"Lund","given":"Jessica M."\},\{"family":"Xavier-Magalhaes","given":"Ana"\},\{"family":"Liang","given":"Yuxin"\},\{"family":"Kurdoglu","given":"Ahmet"\},\{"family":"O’Gorman","given":"William E."\},\{"family":"Modrusan","given":"Zora"\},\{"family":"Le","given":"Daniel"\},\{"family":"Darmanis","given":"Spyros"\}],"accessed":\{"date-parts":[["2026",2,11]]\},"issued":\{"date-parts":[["2024",6,18]]\}\}\}],"schema":"https://github.com/citation-style-language/schema/raw/master/csl-citation.json"\}$(Johnson and Goody 2011; Andrews and Hemberg 2019; Zhang et al. 2019; Simone et al. 2024). In this case, saturation is equal to recovery, and $\hat{L}$ corresponds to the half-saturation constant (i.e. when $R=\hat{L}$, sequencing saturation is 50%):

$E\left[ M \right]=\frac{\hat{L}R}{\hat{L}+R}$ , $Rec=S=100\left( \frac{R}{\hat{L}+R} \right)$

***Fitting the ZT-NB model***

Parameters of the zero-truncated negative binomial (ZT-NB) model were estimated by maximum likelihood using the observed reads-per-molecule (RPM) count histogram. Optimization of the negative log-likelihood was performed using the L-BFGS-B algorithm implemented in scipy.optimize.minimize (SciPy v1.15.3) (Virtanen et al. 2020; Byrd et al. 1995). To reduce sensitivity to local minima, optimization was initialized from multiple starting points spanning a grid of initial parameter values, and the solution with the lowest negative log-likelihood was retained. Model fit was evaluated by comparing the empirical and predicted cumulative distributions using the Kolmogorov–Smirnov (KS) statistic(Wasserman 2010).

***Sample Processing***

*Single Cell RNA-seq Cohorts*

For datasets retrieved from the 10x Genomics website (PBMC and Melanoma samples), BAM files were used when available. If BAM files were not available, the corresponding samples were downloaded and processed using the gene annotation GTF file matching the original Cell Ranger version used to generate the dataset. For samples without BAM files, raw FASTQ files were downloaded and processed with 10x Genomics Cell Ranger v8.0.0 (Zheng et al. 2017).

For the PBMC samples from *Elz et al.* (Elz et al. 2026), FASTQ files were retrieved from the Sequence Read Archive (SRA; BioProject PRJNA1255131) and processed using Cell Ranger v10.0.0 with the GRCh38-2024-A reference. For Flex datasets, the GRCh38-2020-A reference was used together with the matching Human_Transcriptome_Probe_Set_v1.0.1.

Brain snRNA-seq samples from *Maitra et al.* (Maitra et al. 2023) were retrieved from the SRA (BioProject PRJNA883411) and processed using 10x Genomics Cell Ranger v8.0.0 with the GRCh38-2024-A reference. Colon samples from *Elmentaite et al.* (Elmentaite et al. 2021) were retrieved from ArrayExpress (E-MTAB-9543) and processed using 10x Genomics Cell Ranger v8.0.0 with the GRCh38-2024-A reference. Heart samples from *Knight-Schrijver et al.* (Knight-Schrijver et al. 2022) were retrieved from the SRA (BioProject PRJNA891720) and processed using 10x Genomics Cell Ranger v8.0.0 with the GRCh38-2024-A reference. Ovarian cancer samples from *Vázquez-García et al.* (Vázquez-García et al. 2022) were retrieved from the SRA (BioProject PRJNA749041) and processed using 10x Genomics Cell Ranger v8.0.0 with the GRCh38-2024-A reference.

*Visium and Visium HD Cohorts*

Raw FASTQ files were retrieved from the 10x Genomics website and processed using 10x Genomics Space Ranger v4.0.1. Human samples were aligned to the GRCh38-2024-A reference and mouse samples to the mm10-2020-A reference, with the exception of the Visium_HD_Mouse_Brain sample, which required the GRCm39-2024-A reference to match its probe set.

***Calculating countable reads required for a given recovery and saturation***

To convert recovery (as a fraction) to countable reads we use the closed-form solution obtained by solving Equation 3 for $R$.

$$R=\left( \hat{r}\hat{L} \right)\left( \left( 1-Rec/100 \right)^{-1/\hat{r}}-1 \right)$$

To convert a desired saturation value to countable reads we solve the NB-Lib model numerically using a bisection root-finding method. Since the NB-Lib expectation is monotonic in $R$, the root-finding procedure is guaranteed to converge using a bracketing method.

***Alignment tag caching and read classification***

To ensure consistent RPM histogram construction across datasets processed with different reference annotations and software versions, we applied a unified post-alignment filtering and read-classification procedure to all samples.

Basic filtering was applied to all BAM (Li et al. 2009) files requiring a corrected barcode, a raw UMI, and unique mapping. For probe-based assays, the GX tag was used to identify the gene assignment. For other assays, reads were required to map to the correct gene strand, overlap the gene body by at least 95%, and contain a minimum of 40 base pairs of overlap.

Reads were classified based on overlap with annotated exonic and intronic regions, similar to approaches used in RNA velocity methods (He, Soneson, and Patro 2023; Hjörleifsson et al. 2024; La Manno et al. 2018).

- spliced: at least one annotated splice junction
- unspliced: ≥15 bp intronic overlap with no splice junction
- ambiguous: ≥95% of the alignment overlapping exonic regions

Only reads uniquely assigned to a single gene were retained. Tags with identical barcode, gene, and UMI combinations were collapsed and their spliced, ambiguous, and unspliced counts retained. Binary-encoded, compressed, and indexed tag files together with auxiliary metadata files were then generated for each sample.

***Downsampling and Unique molecular identifier collapsing***

To enable deterministic and nested downsampling, each barcode is assigned a unique seed based on a user-defined global seed. Our downsampling framework uses multiprocessing threads to downsample counts associated with barcode. For each alignment tag associated with a given barcode the tags are downsampled using a binomial random number generator and user supplied countable read fractions.

Next, we applied the directional UMI collapsing method by default, while also providing a no-correction option for comparison purposes (Smith, Heger, and Sudbery 2017). We additionally implemented a Cell Ranger–like collapsing strategy in which duplicate UMIs from the same barcode assigned to different genes are assigned to the gene with the highest read count; otherwise, the entire group is discarded.

This approach ensures deterministic nested downsampling across sequencing depths, enabling consistent reuse of read subsets when generating progressively deeper sequencing simulations. The framework also allows robustness to downsampling to be assessed by repeating analyses using multiple global seeds.

Finally, detailed counts and metrics were collected for each barcode, and total, spliced, ambiguous, and unspliced count matrices were generated.

***Cell identification for scRNA-seq cohorts***

A custom python wrapper for emptyDrops (DrupletUtils v1.30.0) (Lun et al. 2019) was implemented within scdepth to generate total count matrices for the full-depth dataset. Cells were further filtered using the maximum of (median - 3 median absolute deviations) or 500 molecules.

***Tissue barcode identification for Visium and Visium HD samples***

We implemented binning in the scdepth package and Visium HD barcodes were binned to 16 x 16$\mu m$ bins (8 x 8 barcodes). The 10x Genomics Space Ranger in_tissue annotations were then used to identify bins located within tissue regions. A molecular cutoff was defined as the maximum of (median molecule count - 3 median absolute deviations) or 500 molecules. For Visium HD samples, we used the maximum of four median absolute deviations or 100 molecules.

***Limited saturation analysis***

The NB-Lib framework was used to estimate the number of countable reads required to reach a desired saturation level. The scdepth framework was then used to downsample each sample to the corresponding countable read fractions.

*Preseq benchmarking*

The RPM histograms produced by scdepth were used as input for preseq v3.2.0 (Daley and Smith 2013) for sequencing curve estimation, enabling direct comparison between NB-Lib and preseq using the same input data.

*preseq lc_extrap -Q -H <RPM histogram> -e <max reads target> -s <read step> -o <out>*

If the command failed, we also tried running it with the *-D* option. The resulting curves were then interpolated to the required countable read depths using NumPy v2.2.6.

For library complexity analyses we used

*preseq bound_pop -H <hist> -o <out>*

***Gene stability profiling***

For samples with at least 70% molecular recovery, we used our downsampling framework to quantify gene detection stability across decreasing recovery levels. At each downsampling step, detected genes for each barcode were compared to those detected at full sequencing depth. Gene stability was defined as the fraction of genes detected at full sequencing depth that remained detected in the corresponding downsampled barcode. Genes were stratified for each barcode based on their molecule counts at full depth into four groups: 1 molecule, 2 molecules, 3 or more molecules, and a global gene set including all detected genes. Barcodes were included for each class if they had at least 10 unique gene observations. For each sample, 10 downsampling seeds were used. Stability was first summarized per barcode by taking the median across seeds and then summarized at the sample level by taking the median across all barcodes.

***Sample clustering***

scdepth was used to generate total count matrices at each recovery level and at full dataset depth. Scanpy v1.11.5(Wolf, Angerer, and Theis 2018) was used for downstream processing and clustering. Genes were filtered using scanpy.pp.filter_genes with min_cells=10. Next, 2,000 highly variable genes were selected using scanpy.pp.highly_variable_genes with flavor='seurat_v3'. Counts were then normalized to a total of 10,000 counts per cell and log-transformed using log1p. Principal component analysis was performed using 30 principal components, followed by construction of a 30-nearest-neighbor graph. Leiden clustering was then performed at 0.6, 0.8, and 1.0 resolution.

To assess clustering stability across downsampling seeds, we evaluated several metrics. Highly variable gene (HVG) stability was quantified as the median pairwise Jaccard similarity between HVG sets across seeds. Similarly, K-nearest-neighbor (KNN) stability was quantified as the pairwise Jaccard similarity between KNN graphs for each barcode across seeds. Finally, clustering stability was assessed using the adjusted Rand index (Hubert and Arabie 1985) (ARI) calculated between cluster label assignments across seeds.

***References***

Andrews, Tallulah S., and Martin Hemberg (2019), ‘M3Drop: Dropout-Based Feature Selection for scRNASeq’, *Bioinformatics*, 35/16: 2865–7, https://doi.org/10.1093/bioinformatics/bty1044.

Byrd, Richard H., et al. (1995), ‘A Limited Memory Algorithm for Bound Constrained Optimization’, *SIAM Journal on Scientific Computing*, 16/5: 1190–208, https://doi.org/10.1137/0916069.

Elmentaite, Rasa, et al. (2021), ‘Cells of the Human Intestinal Tract Mapped across Space and Time’, *Nature*, 597/7875: 250–5, https://doi.org/10.1038/s41586-021-03852-1.

Elz, Anna E., et al. (2026), ‘Evaluating the Practical Aspects and Performance of Commercial Single-Cell RNA Sequencing Technologies’, *NAR Genomics and Bioinformatics*, 8/1: lqaf215, https://doi.org/10.1093/nargab/lqaf215.

He, Dongze, Charlotte Soneson, and Rob Patro (2023), ‘Understanding and Evaluating Ambiguity in Single-Cell and Single-Nucleus RNA-Sequencing’, preprint, bioRxiv, 4 January, 2023.01.04.522742, https://doi.org/10.1101/2023.01.04.522742.

Hilbe, Joseph M. (2014), *Modeling Count Data* (Cambridge), https://doi.org/10.1017/CBO9781139236065.

Hjörleifsson, Kristján Eldjárn, et al. (2024), ‘Accurate Quantification of Single-Cell and Single-Nucleus RNA-Seq Transcripts Using Distinguishing Flanking k-Mers’, preprint, bioRxiv, 26 January, 2022.12.02.518832, https://doi.org/10.1101/2022.12.02.518832.

Johnson, Kenneth A., and Roger S. Goody (2011), ‘The Original Michaelis Constant: Translation of the 1913 Michaelis–Menten Paper’, *Biochemistry*, 50/39: 8264–9, https://doi.org/10.1021/bi201284u.

Knight-Schrijver, Vincent R., et al. (2022), ‘A Single-Cell Comparison of Adult and Fetal Human Epicardium Defines the Age-Associated Changes in Epicardial Activity’, *Nature Cardiovascular Research*, 1/12: 1215–29, https://doi.org/10.1038/s44161-022-00183-w.

La Manno, Gioele, et al. (2018), ‘RNA Velocity of Single Cells’, *Nature*, 560/7719: 494–8, https://doi.org/10.1038/s41586-018-0414-6.

Li, Heng, et al. (2009), ‘The Sequence Alignment/Map Format and SAMtools’, *Bioinformatics*, 25/16: 2078–9, https://doi.org/10.1093/bioinformatics/btp352.

Maitra, Malosree, et al. (2023), ‘Cell Type Specific Transcriptomic Differences in Depression Show Similar Patterns between Males and Females but Implicate Distinct Cell Types and Genes’, *Nature Communications*, 14/1: 2912, https://doi.org/10.1038/s41467-023-38530-5.

Simone, Marco De, et al. (2024), ‘Comparative Analysis of Commercial Single-Cell RNA Sequencing Technologies’, preprint, bioRxiv, 18 June, 2024.06.18.599579, https://doi.org/10.1101/2024.06.18.599579.

Smith, Tom, Andreas Heger, and Ian Sudbery (2017), ‘UMI-Tools: Modeling Sequencing Errors in Unique Molecular Identifiers to Improve Quantification Accuracy’, *Genome Research*, 27/3: 491–9, https://doi.org/10.1101/gr.209601.116.

Vázquez-García, Ignacio, et al. (2022), ‘Ovarian Cancer Mutational Processes Drive Site-Specific Immune Evasion’, *Nature*, 612/7941: 778–86, https://doi.org/10.1038/s41586-022-05496-1.

Virtanen, Pauli, et al. (2020), ‘SciPy 1.0: Fundamental Algorithms for Scientific Computing in Python’, *Nature Methods*, 17/3: 261–72, https://doi.org/10.1038/s41592-019-0686-2.

Wasserman, Larry (2010), *All of Statistics : A Concise Course in Statistical Inference* (New York), http://www.amazon.de/All-Statistics-Statistical-Inference-Springer/dp/1441923225/ref=sr_1_2?ie=UTF8&qid=1356099149&sr=8-2.

Zhang, Xiannian, et al. (2019), ‘Comparative Analysis of Droplet-Based Ultra-High-Throughput Single-Cell RNA-Seq Systems’, *Molecular Cell*, 73/1: 130-142.e5, https://doi.org/10.1016/j.molcel.2018.10.020.

Zheng, Grace X. Y., et al. (2017), ‘Massively Parallel Digital Transcriptional Profiling of Single Cells’, *Nature Communications*, 8/1: 14049, https://doi.org/10.1038/ncomms14049.
