# Supplementary material for: Sequencing saturation does not uniquely determine molecular recovery in UMI transcriptomics
Source: Bioinformatics. 2026 Aug 8;42(8):btag593. doi: 10.1093/bioinformatics/btag593 (PMC13476072; doi:10.1093/bioinformatics/btag593)
Supplement: Supplementary file 1 [file btag593_supplementary_data.zip › Supplemental/Supplemental Note 2.docx]

**Methods:**

*Parse WT sample processing*

Raw sequencing reads were downloaded from the Sequence Read Archive (SRA) using the following accessions: SRR33305100, SRR33305102, SRR33305104, SRR33305106, SRR33305101, SRR33305103, SRR33305105, SRR33305107

SRA Lite files were converted to FASTQ format using fasterq-dump. Reads were processed with Parse Biosciences Pipeline v1.8.1 using the Cell Ranger GRCh38-2024-A (hg38) gene annotation to maintain consistency with the matched 10x Genomics samples. The resulting BAM file was used as input for scdepth.

*Tag caching and curve fitting*

The BAM file was first processed together with multiplexed sample metadata. Compared to the default settings, the minimum number of gene-aligned bases required for tag assignment (--min-gene-bases) was reduced from 40 to 30. We also added support to parse the originating primer (poly(A) or random hexamer) from the read query name, allowing each tag to be annotated with its primer of origin.

scdepth cache --min-gene-bases 30 -g genes_fixed.gtf.gz -t 6 -l Parse_WT_v3 --sample-tag pS --samples B2-PA-F1A,B2-PA-F1B,B2-PA-F5A,B2-PA-F5B barcode_headAligned_anno.bam Parse_WT_PBMC/scdepth

EmptyDrops (Lun et al. 2019) was then run independently for each sample, with samples inferred automatically from the multiplexed metadata. Barcodes were further filtered as described in the Methods.

scdepth emptydrops -t 6 -s Parse_WT_PBMC Parse_WT_PBMC/scdepth

Finally, a single baseline sequencing-depth curve was fit across the entire dataset for subsequent downsampling analyses.

scdepth fit -t 6 -s Parse_WT_PBMC Parse_WT_PBMC/scdepth

*Parse WT sample analyses*

All analyses presented for the Parse WT sample were performed using code available in the scdepth_manuscript GitHub repository (notebooks/Parse WT.ipynb).

**Results:**

***NB-Lib accurately models the RPM distributions for a Parse WT v3 Library***

The Parse Biosciences WT v3 library was downloaded from the Sequence Read Archive (SRA) and processed as described in the Methods. The library contained four multiplexed samples comprising two biological replicates (F1 and F5), each with two technical replicates (A and B). These samples were matched to the corresponding 10x Genomics v3.1, v4, and Flex libraries from *Elz et al*. (Elz et al. 2026). The library contained 1.48 billion total sequencing reads, of which 1.02 billion were countable, with an overall sequencing saturation of 55.3%. Following EmptyDrops and quality-control filtering, the four samples contained 21,579 (F1A), 15,412 (F1B), 21,044 (F5A), and 17,243 (F5B) cells.

To evaluate the applicability of NB-Lib to a Parse Biosciences WT v3 library, we analyzed the combined multiplexed library containing all four samples and both primer types, as this represents the intended use case for sequencing depth planning and library-level analyses because sequencing depth planning is performed at the level of the prepared sequencing library. We evaluated the quality of the fit using our baseline curve estimation (See Methods) by downsampling the library to 50% saturation and observed a good ZT-NB fit to the reads-per-molecule (RPM) distribution with a Kolmogorov–Smirnov (KS) statistic of 0.0036 (**Fig.S9A**). Using NB-Lib, we estimated the number of countable reads required to achieve a range of saturation values and validated the predictions using scdepth. Across saturation values from 10% to 55%, the mean absolute error (MAE) was 0.07 percentage points (pp), demonstrating excellent agreement between predicted and observed values and (**Fig. S9B**). These results are comparable to the KS statistics and saturation prediction errors observed across the primary 10x Genomics cohorts. Next, we performed limiting saturation analyses and observed a small increase in KS and a consistent MAE across saturation values ranging from 10 to 55% (**Fig.S9 C,D)**. Although the library reached only 55% saturation at full sequencing depth, limiting the range of evaluated saturation values, the results remained consistent with accurate saturation prediction. Finally, we determined the minimum countable reads per cell for reliable saturation curve estimation of ~500-1000 reads per cell similar to the results we observed with 10X Genomics scRNA-seq libraries (**Fig. S9E-G)**.

Parse WT libraries are multiplexed and contain multiple samples together with two reverse transcription primer types, poly(dT) (poly(A)) and random hexamers. To evaluate whether sample multiplexing and primer type influence NB-Lib parameter estimation, we implemented support in **scdepth** to retain sample and primer assignments throughout molecule counting and downsampling. The full Parse WT v3 library was downsampled to saturation values from 10% to 55% in 5% increments, after which sub-library RPM histograms, ZT-NB fits, and NB-Lib statistics were calculated.

We first observed that although the number of reads allocated to each sub-library differed, the resulting saturation curves were nearly identical (Fig. S10A,B). Likewise, the ZT-NB goodness-of-fit (KS statistic) and estimated amplification heterogeneity parameter ($\hat{r}$), were highly consistent across the combined library, individual samples, and primer-specific subsets (Fig. S10C–D). Finally, normalized library complexity ($\hat{L}$ per cell), and saturation prediction was also similar across sub-libraries, and the saturation MAE remained low and comparable to that observed for the full Parse WT v3 library (**Fig. S10E,F**).

**References:**

Elz, Anna E., et al. (2026), ‘Evaluating the Practical Aspects and Performance of Commercial Single-Cell RNA Sequencing Technologies’, *NAR Genomics and Bioinformatics*, 8/1: lqaf215, https://doi.org/10.1093/nargab/lqaf215.

Lun, Aaron T. L., et al. (2019), ‘EmptyDrops: Distinguishing Cells from Empty Droplets in Droplet-Based Single-Cell RNA Sequencing Data’, *Genome Biology*, 20/1: 63, https://doi.org/10.1186/s13059-019-1662-y.
